# Neonicotinoid Insecticides and Their Impacts on Bees: A Systematic Review of Research Approaches and Identification of Knowledge Gaps

**DOI:** 10.1371/journal.pone.0136928

**Published:** 2015-08-27

**Authors:** Ola Lundin, Maj Rundlöf, Henrik G. Smith, Ingemar Fries, Riccardo Bommarco

**Affiliations:** 1 Swedish University of Agricultural Sciences, Department of Ecology, SE-750 07 Uppsala, Sweden; 2 University of California, Department of Entomology and Nematology, Davis, California 95616, United States of America; 3 Lund University, Department of Biology, SE-223 62 Lund, Sweden; 4 Lund University, Centre for Environmental and Climate Research, SE-223 62 Lund, Sweden; University of Guelph, CANADA

## Abstract

It has been suggested that the widespread use of neonicotinoid insecticides threatens bees, but research on this topic has been surrounded by controversy. In order to synthesize which research approaches have been used to examine the effect of neonicotinoids on bees and to identify knowledge gaps, we systematically reviewed research on this subject that was available on the Web of Science and PubMed in June 2015. Most of the 216 primary research studies were conducted in Europe or North America (82%), involved the neonicotinoid imidacloprid (78%), and concerned the western honey bee *Apis mellifera* (75%). Thus, little seems to be known about neonicotinoids and bees in areas outside Europe and North America. Furthermore, because there is considerable variation in ecological traits among bee taxa, studies on honey bees are not likely to fully predict impacts of neonicotinoids on other species. Studies on crops were dominated by seed-treated maize, oilseed rape (canola) and sunflower, whereas less is known about potential side effects on bees from the use of other application methods on insect pollinated fruit and vegetable crops, or on lawns and ornamental plants. Laboratory approaches were most common, and we suggest that their capability to infer real-world consequences are improved when combined with information from field studies about realistic exposures to neonicotinoids. Studies using field approaches often examined only bee exposure to neonicotinoids and more field studies are needed that measure impacts of exposure. Most studies measured effects on individual bees. We suggest that effects on the individual bee should be linked to both mechanisms at the sub-individual level and also to the consequences for the colony and wider bee populations. As bees are increasingly facing multiple interacting pressures future research needs to clarify the role of neonicotinoids in relative to other drivers of bee declines.

## Introduction

Animal pollination, mainly performed by bees, is an important ecosystem service with almost 90 percent of flowering plants and 75 percent of the world’s most common crops benefiting from animal flower visitation [1–2]. Habitat loss and fragmentation, pesticides, pathogens, climate change, invasive species, intense management of managed bees, and decreased interest in beekeeping have all been suggested as threats to bees and pollination services, but the relative importance of these drivers remains uncertain [3–4].

More recently, the use of neonicotinoid insecticides has been specifically pointed out as a factor that might contribute to declines of both managed and wild bees [5–6]. Neonicotinoid compounds are used in more than 120 countries with at least 140 different crop uses (e.g. soil and foliar applications of the same compound in the same crop are defined as two different crop uses) [7]. Since their commercial introduction in the early 1990s, neonicotinoids have quickly become the most commonly used class of insecticides in the world. Their market share grew rapidly from 16 percent in 2005 to 24 percent in 2008, valued at roughly €1.5 billion in 2008 [7–8]. Neonicotinoids have high selectivity towards invertebrate over vertebrate organisms [9]. They are taken up systemically and can be present in all plant tissues, which makes them efficient against a wide range of pests over a protracted time period and when applied in small quantities, e.g. as seed treatments [7–9]. At the same time, several of the neonicotinoid compounds have been shown to be highly toxic to bees in very small quantities [10]. However, with the exception of exposure to dust emission from pneumatic seeders during sowing of treated seeds [11], estimates of bees’ exposure to neonicotinoids generally are substantially lower than levels causing acute mortality. Neonicotinoids can be translocated into pollen and nectar, the principal food sources for bees [12]. Moreover, some of the compounds degrade slowly and are present in the environment, e.g. in soil and/or treated plants for months, or even years, after the application [6,13–14]. Concern for pollinators has led to a temporary restriction of three neonicotinoids (clothianidin, thiamethoxam and imidacloprid) as seed treatments for use on crops attractive to bees in the European Union [15] and a policy to reduce the use of the same three insecticides as seed treatments for maize and soy by 80 percent from 2014 levels in Ontario, Canada [16]. However, significant knowledge gaps and controversy remain as to whether such restrictions are justified [17–19].

Reviews on the effect of neonicotinoids on bees have so far often dealt either specifically with the role of neonicotinoids for honey bee declines or colony losses [20–24], or more broadly with the effects of neonicotinoid use on the wider environment [6]. The most comprehensive reviews on neonicotinoid effects on bees have focused on concentrations of neonicotinoids found in the environment that bees might be exposed to, effects of neonicotinoids on bees, and risk assessment [5,25–26]. Additionally, the status of the natural science evidence base for effects of neonicotinoids on bees has recently been summarized [27]. In this study we examine which research approaches have been used in this area by conducting a systematic review of the literature. More specifically, for each study reviewed we asked the following questions: (i) from which country did the study originate, (ii) which neonicotinoid compounds were studied, (iii) which crops were studied, (iv) which bee species were studied, (v) which methodological approaches were used, and (vi) what biological levels, from the sub-individual to the population level, were studied? By asking these questions we aimed to systematically characterize how our current knowledge about the effect of neonicotinoids on bees has been derived and to identify research gaps that can be addressed in future studies.

## Methods

We searched in the Web of Science Core Collection and PubMed for studies that had examined the effects of neonicotinoids on bees (last access date: 20 June 2015). The search was limited to the Web of Science and PubMed because it contained research articles that were available in full text, written in English, and that had undergone peer-review by scientists. We used the following search string to locate potential studies on neonicotinoids and bees: (neonic* OR imidacloprid OR clothianidin OR thiamethoxam OR acetamiprid OR thiacloprid OR nitenpyram OR dinotefuran) AND (*bee OR *bees).

The primary database consisted of 543 publications, from which we removed duplicates. Conference proceedings and book chapters that appeared in the search were excluded from further review to ensure easy access to full text publications. Publications not written in English were also excluded. Remaining database records were retrieved in full text and inspected in detail. For primary research articles to be included in our review they had to contain either a measure of an effect of a neonicotinoid on bees, or a measure of neonicotinoid contamination of bees or plants, hive products or other material that bees come into contact with or digest. Article types other than primary research (see categories below) were also included if they dealt with these topics. Publications in analytical chemistry that focused on developing methods to analyze neonicotinoids were included only if they included determinations of neonicotinoids in the natural environment.

Each publication included in the study was reviewed using a standard review protocol, with information collected as described below. We collected full reference information and extracted information about whether the publication was primary research, a review, meta-analysis, or another type of article (e.g. comment, opinion, essay or editorial). As our review questions were designed for primary research publications, we further evaluated only these publications. For each primary research study we noted the country where the study was performed, focal neonicotinoid compound(s), crop species and application methods examined in each crop studied, and bee species. For studies that lacked information about where the research was performed, as was the case for some laboratory studies, we used the location of the first author’s institution. A focal crop species was only noted if measurements were made in that crop, or if bees were exposed to that crop during the study. Hence, a focal crop was noted for laboratory studies only when it was included in the experiment, but not when the experimental treatment mimicked the application details for a certain crop. Each study could include zero to several focal crops and bee species, and one to several focal neonicotinoid compounds.

The methods used in primary research studies were categorized into five different methodological approaches: laboratory, semi-field, field, *in silico*, or combined approach. Studies in which the treatments and data collection were conducted in the laboratory or greenhouse were jointly classified into “laboratory approach”. Designs that used cages or tunnels in the field were classified as “semi-field approach”. “*In silico* approach” included modeling or risk assessments. “Combined approach” used more than one approach for single endpoints. Examples include combined laboratory and field designs, where the treatment applied to the study subjects was performed in the field, and the effects were observed in the laboratory on the same study subjects, or vice versa. Most studies could be assigned to a single methodological approach, but some studies that used different approaches for different endpoints were classified into multiple categories. For example, publications in which some endpoints were measured on one set of study subjects in a laboratory experiment, and another set of endpoints were measured on different set of study subjects in the field.

Finally, we mapped at what level of biological organization each study measured effects. Endpoint measurements of each study were grouped into four classes: (i) sub-individual measure (e.g. gene expression, cell death, neurotransmission, or physiological measures), (ii) individual measure (e.g. learning, memory, movement, or mortality), (iii) colony measure (e.g. colony mass, colony reproduction, or colony survival), or (iv) population measure (e.g. bee population size).

**Fig 1:**
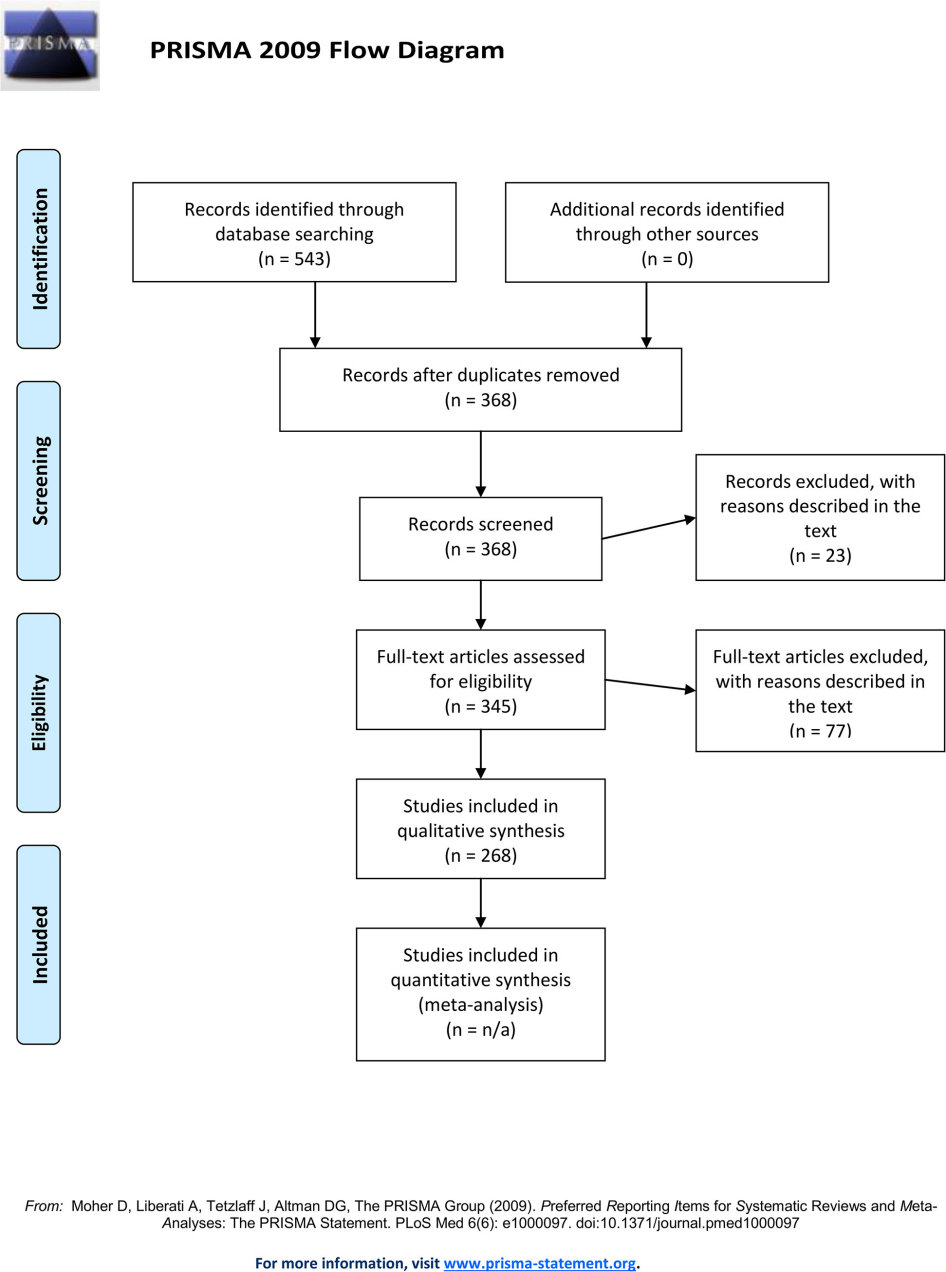
Flow diagram for the systematic review.

Fig 1 depicts a flow diagram for the systematic review. A checklist for the systematic review can be found in the Supplementary Information (S1 PRISMA Checklist).

## Results

### Article types and publication years

**Fig 2:**
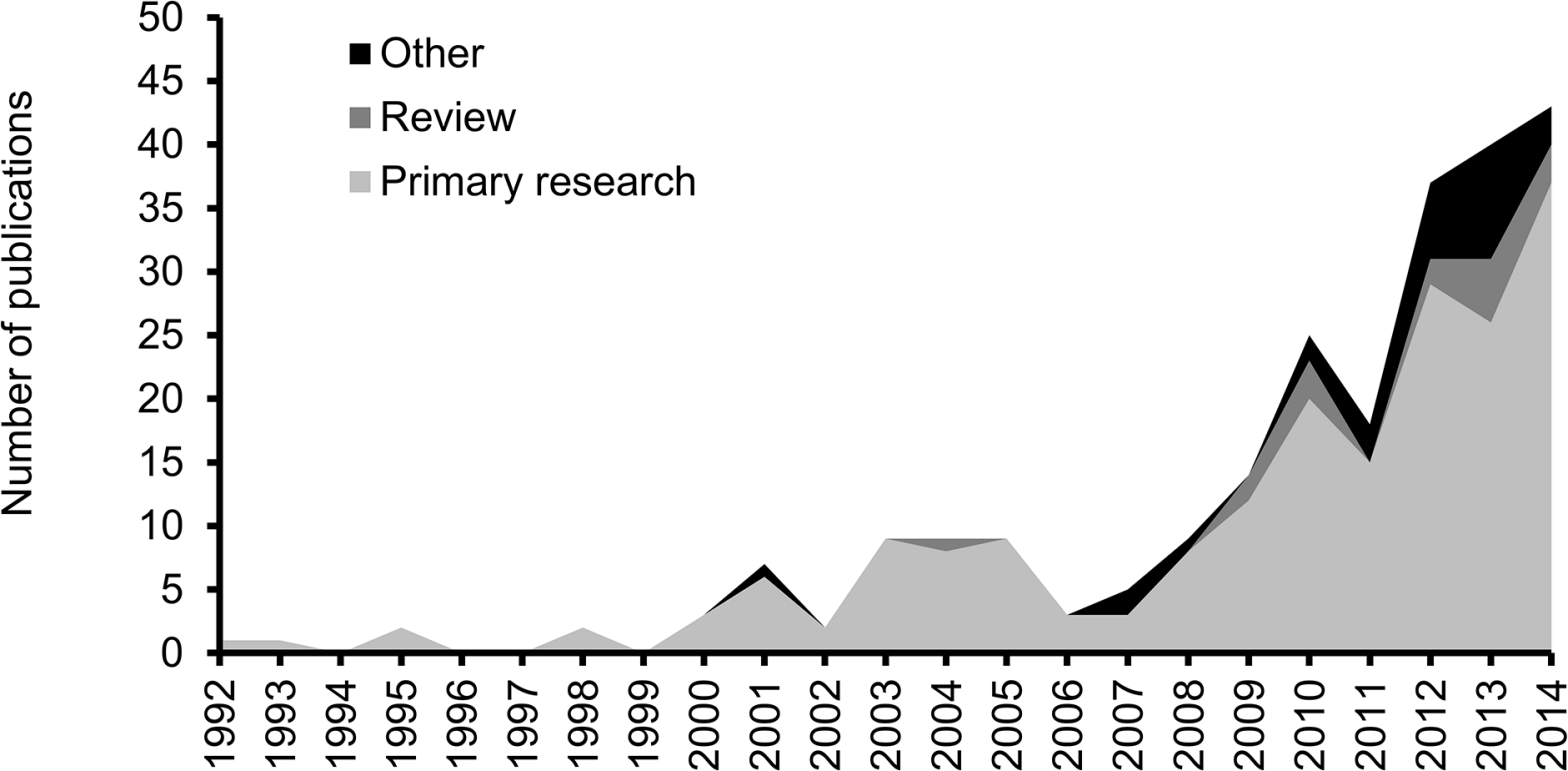
Development of research on the effect of neonicotinoids on bees over time. The single meta-analysis study was published in 2011 [12] and is not included in this figure. Data for 2015 (not complete; see text) included 20 primary research publications, 2 reviews and 6 other publications (not included in figure).

A total of 268 publications matched our criteria; 216 were primary research, 18 were reviews, one was a meta-analysis, and 33 were other publication types. Full references for all publications and data for each primary research publication are presented in S1 Table. Approximately half of the studies were published within the last three years, demonstrating a rapid expansion of the research field (Fig 2).

#### Geographical distribution of studies

**Fig 3:**
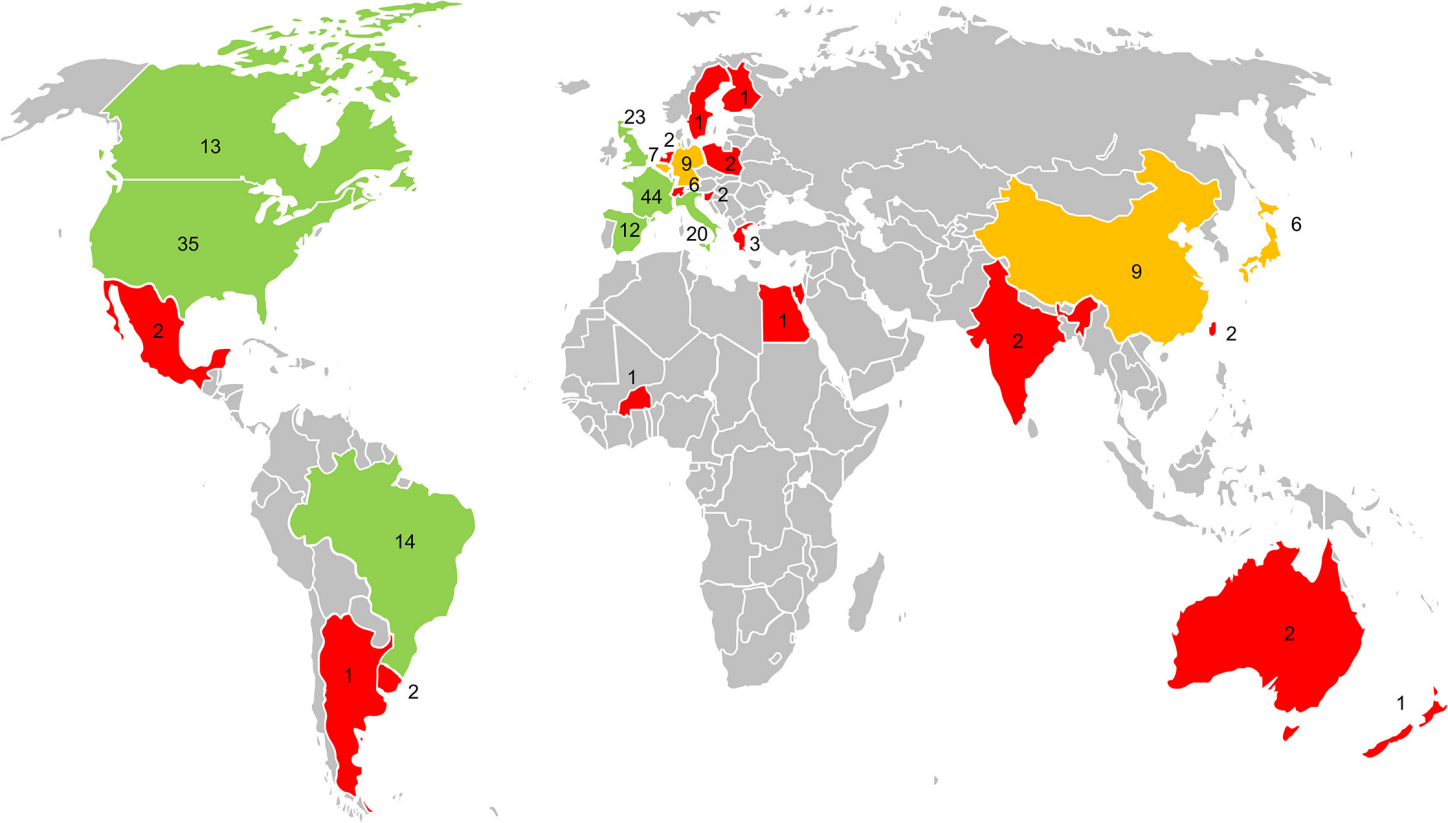
Geographical distribution of research on neonicotinoid impacts on bees. The number of primary research studies from each country is indicated. Colours indicate countries with 1–5 studies (red), 6–10 studies (orange) or more than 10 studies (green).

Primary research studies were conducted in 27 countries. However, more than half of the studies were from four countries: France (n = 44), the United States (n = 35), the United Kingdom (n = 23) and Italy (n = 20). Overall, 82 percent of the studies were done in Europe or North America (Fig 3). Nine percent of the studies were from Asia (n = 19) and 8 percent were from South America (n = 17). We found three studies from Oceania and two studies from Africa.

### Neonicotinoid compounds

Imidacloprid was the most commonly studied compound (included in 78 percent of studies, n = 168), followed by thiamethoxam (34 percent, n = 73), clothianidin (33 percent, n = 71), acetamiprid (19 percent, n = 40), thiacloprid (18 percent, n = 39), dinotefuran (7 percent, n = 15), and nitenpyram (6 percent, n = 13).

### Crop species

Maize was the most commonly studied crop (28 studies), followed by oilseed rape (canola: 7 studies) and sunflower (7 studies). Between one and four studies were found for each of 13 other crops (Table 1).

**Table 1:** Total number of studies on neonicotinoids and bees in different crops, study examples for each crop, and number of studies for each method of application in each crop (‘Seed’ = seed treatment application, ‘Foliar’ = foliar spray application, ‘Soil’ = furrow, drench or drip irrigation application, Granulate = granulate application).

Crop Linnean name	Common name	# studies	Study example	Application method			
				Seed	Foliar	Soil	Granulate
*Zea mays*	Maize	28	[28]	28			
*Brassica napus*	Oilseed rape	7	[29]	6	3		
*Helianthus annuus*	Sunflower	7	[30]	7			
*-*	Turfgrass	4	[31]		4		1
*Cucumis melo*	Cantaloupe	3	[32]		1	2	
*Gossypium spp*.	Cotton	3	[33]	1	2		
*Solanum lycopersicum*	Tomato	3	[34]		2	2	
*Citrus spp*.	Citrus fruits	2	[35]		1	1	
*Cucurbita pepo*	Pumpkin, squash	2	[36]	1	1	2	
*Malus domestica*	Apple	2	[37]		2		
*Brassica juncea*	Mustard	2	[38]	1	1		
*Actinidia spp*.	Kiwifruit	1	[39]				
*Brassica rapa*	Turnip rape	1	[40]		1		
*Glycine max*	Soybean	1	[33]	1			
*Medicago sativa*	Alfalfa	1	[10]		1		
*Triticum spp*.	Wheat	1	[41]	1			

### Bee species

The western honey bee *Apis mellifera* was the most common bee species studied (162 studies) followed by the two bumble bee species *Bombus terrestris* (24 studies) and *Bombus impatiens* (10 studies). Between one and six studies were found for each of 15 other bee species or species groups (Table 2).

**Table 2:** Number of studies examining the effect of neonicotinoids on different bee species.

Species	# studies
*Apis mellifera*	162
*Bombus terrestris*	24
*Bombus impatiens*	10
*Apis cerana*	6
*Bombus spp*.	4
*Megachile rotundata*	3
*Apoidea spp*.	3
*Melipona quadrifasciata*	3
*Osmia bicornis*	2
*Osmia lignaria*	2
*Bombus hypocrita*	1
*Bombus ignitus*	1
*Bombus occidentalis*	1
*Bombus patagiatus*	1
*Nannotrigona perilampoides*	1
*Nomia melanderi*	1
*Osmia cornifrons*	1
*Scaptotrigona postica*	1

### Methodological approaches

A total of 112 studies used laboratory approaches, 92 used field approaches, 14 used semi-field approaches, and 12 used *in silico* approaches. Twenty-five studies used combined approaches. They most often combined laboratory and field approaches where the treatment was applied in the field and the effects were observed in the laboratory or vice versa. There were more methodological approaches than primary research studies because 32 studies used more than one approach (see S1 Table).

### Biological levels–from sub-individual to population effects

Most studies measured the effects of neonicotinoids on bees at the individual level (n = 109 studies), followed by the colony level (n = 60) and the sub-individual level (n = 48). None of the studies investigated effects on the population level according to our definitions.

## Discussion

### Geographical distribution of studies

We found that most studies originated from just a few countries in Europe and North America. The skewed geographical distribution of studies is potentially problematic, especially when it comes to the paucity of field studies from countries outside Europe and North America. The use of neonicotinoids is geographically widespread: for example, imidacloprid has been registered for use in at least 120 countries [7]. Although more detailed global records of neonicotinoid use around the world are lacking, insecticide use is generally more intense in upper-middle income countries compared to high income countries, primarily in Europe and North America [42]. Furthermore, pesticides are also generally more weakly regulated in countries outside Europe and North America [42]. Finally, areas outside Europe and North America host the majority of the global crop pollination value. It has been estimated that 58 percent of the global economic value of insect pollination originates from Asia, with another 8 percent originating from Africa and a further 10 percent from South and Central America [43]. These factors suggest the need to assess the impacts of neonicotinoid use on bees and crop pollination services in countries outside Europe and North America. It is, however, important to acknowledge that our method of searching for studies only in the Web of Science and PubMed might have skewed results towards finding more studies from Europe and North America, and that alternative searches using Google Scholar, for example, may reveal additional studies from other countries.

### Neonicotinoid compounds

When the number of studies we found for each neonicotinoid compound is compared against global sales data from 2009 (available in [7]) it can be concluded that there is a positive relationship between these two variables. Typically neonicotinoid compounds with higher sales figures have also attracted a greater number of studies. Thiamethoxam deviates the most from this relationship and has been studied less than would be expected from sales data. Imidacloprid, thiamethoxam and clothianidin are all highly toxic to honey bees (acute oral LD_50_ for *A*. *mellifera*: 0.004–0.005 μg per bee, acute contact LD_50_ for *A*. *mellifera*: 0.02–0.08 μg per bee [44]), although these quantities still are appreciably higher than those typically encountered by bees in the environment. They also have similar application methods and crop uses [7]. It is thus unlikely that the greater attention that imidacloprid and clothianidin have received relative to their market shares are due to bees being less at risk of being exposed to, and affected by, thiamethoxam. We conclude that more studies on thiamethoxam are warranted. Similarly, there is a need for comparative studies that assess how well the large numbers of studies on imidacloprid reflect the effects of neonicotinoid compounds as a group.

On the other hand, acetamiprid and thiacloprid, are several orders of magnitude less toxic to honey bees compared to the other neonicotinoids (acute oral LD_50_ for *A*. *mellifera*: 15–17 μg per bee, acute contact LD_50_ for *A*. *mellifera*: 8.1–39 μg per bee) [44]. A similar story seems to be true for the persistence of these compounds as thiacloprid and acetamiprid have shorter half-lives in the environment (3–74 days in soil for thiacloprid, 31–450 for acetamiprid) compared to imidacloprid (28–1250 days), clothianidin (148–6931 days) and thiamethoxam (7–353 days, clothianidin is a primary metabolite) [6]. The comparatively more “bee-friendly” properties of these compounds have, however, led to more liberal usage criteria: for instance, these chemicals are sometimes permitted for use on flowering crops [27]. In the case of the insecticide Biscaya OD 240, containing the active ingredient thiacloprid, the Swedish product information states that it can be used during daylight in flowering crops [45] when bees will be actively foraging. To our knowledge there have been few studies assessing the risks associated with such applications for bees and crop pollination (but see [46]), and this is an area that deserves further attention.

### Crop species

Neonicotinoids are used in a wide number of crops, including many fruit and vegetables [8] that often are dependent on pollinators for yield, and are highly attractive to bees [1]. Imidacloprid has at least 140 different crop uses registered [7]. Despite this, we found that research about neonicotinoid impacts on bees has mainly focused on only three, albeit large, crop uses, namely seed treatments in maize, oilseed rape and sunflower.

The most common crop studied was maize (28 studies). These studies primarily investigated potential effects on honey bees resulting from (i) dust from seeds coated with neonicotinoids during sowing (e.g. [28]), (ii) guttation drops formed on treated plants (e.g. [47]), and (iii) pollen collection by bees from seed treated maize (e.g. [48]). In the seven studies each on oilseed rape and sunflowers, the focus was on potential sublethal effects on bees resulting from movement/transfer of neonicotinoids from seed treatments into pollen and nectar [29–30].

Only a few studies investigated fruit or vegetable crops, and most of them examined only the potential for neonicotinoid exposure to bees. For example, soil applications result in markedly higher neonicotinoid residue levels in the pollen and nectar of pumpkin and squash compared with seed treated maize, oilseed rape and sunflower [36,49]. This is an important finding because decisions of what constitutes field-realistic exposure for bees in experiments (e.g. [50–53]) and risk assessment studies ([54–55] but see [56]) are often based on information from seed treated maize, oilseed rape and sunflower. If it turns out that residue levels in the pollen and nectar of these crops (maize, oilseed rape and sunflower) are not representative of other crops, the hazard that neonicotinoids pose for bees might be incorrectly estimated.

Little attention has been given to neonicotinoid use on plants other than field crops, such as lawns with flowering weeds (but see [31,57–59]), fruit trees, home garden plants, ornamental plants, bushes and trees. Information available from studies not included on either Web of Science or PubMed [13], as well as from a recent study on pesticide residues in pollen and nectar from citrus trees treated with imidacloprid [35], suggests that neonicotinoids can be present at higher levels for an extended period of time in woody plants compared to non-woody plants. It was therefore surprising that exposure to bees from neonicotinoid treated woody plants, such as pome fruit, stone fruit and citrus fruit trees, and on ornamental and garden plants [7], has been largely unstudied. Future research should be conducted on a wider array of neonicotinoid treated agricultural crops, including methods of application other than seed treatment, and should more comprehensively assess the consequences for bees of neonicotinoid use on other plants.

### Bee species

Most studies (75 percent) included measures of neonicotinoid exposure or effects on *A*. *mellifera*. Although the second and third most commonly studied species, the bumble bees *B*. *terrestris* and *B*. *impatiens*, were included in only eleven and five percent of studies respectively, they were still studied substantially more than any other bee species. Very few studies have examined the effect of neonicotinoids on solitary bees or social bees other than honey bees or bumble bees (e.g. stingless bees [60]).

An interesting question is whether this knowledge gap for bee species other than *A*. *mellifera* may be overcome by extrapolating information from honey bees to other bees. Comparative studies in which the same experimental protocols are applied to *A*. *mellifera* and other bee taxa can help answer this question. The few studies that have done this for neonicotinoids have found different responses among bee species [61–64]. However, results from a meta-analysis suggest there is a general positive correlation between the lethal toxicity of pesticides for *A*. *mellifera* and other bee species [65]. Part of the variation in pesticide toxicity among species might be explained by differences in body size, with larger bee species generally showing less sensitivity to a certain pesticide treatment [65–66]. However, this scope for predictability across bee species considers only lethal pesticide toxicity at the level of the individual bee. These trends may not hold true for the sublethal effects of neonicotinoid exposure. For example, *B*. *terrestris* has been found to be more sensitive than *A*. *mellifera* for some endpoints, even though the former has a larger body size [62,64]. In addition, there is a substantial variation in ecological, phenological and life-history traits among bee species, such as in preferred nesting habitats, flight seasons and degree of sociality. This implies that both the exposure to neonicotinoids and other pesticides, and their impacts at the colony and population levels, will vary significantly depending on the traits of the bee species [50,67–69]. For these reasons, it is unlikely that studies on *A*. *mellifera* can fully explain and/or predict the effects of neonicotinoids on other bee species.

*A*. *mellifera* is an important pollinator of many crops [1]. However, wild bees provide a complement to honey bees as pollinators, and the contribution of wild bees to crop pollination has likely been underestimated [70–71]. The reliance on the pollination services from wild bees might also be increasing as crop pollination demands are growing much faster than honey bee stocks [72–73], and wild bees may provide insurance against honey bee declines [74–75]. Future studies should therefore verify that neonicotinoid use does not jeopardize pollination across the wide range of bee species that deliver these essential ecosystem services.

### Methodological approaches

Laboratory studies were most common. They are valuable for detecting the mechanisms of neonicotinoid impacts on bees [27]. Many laboratory studies have so far investigated the effects of neonicotinoids at concentrations above the field-realistic range [12]. This is a reasonable first step to verify if any effect is detectable. However, subsequent laboratory studies to investigate an effect that is already known to occur can be significantly improved by testing more realistic exposure scenarios. Increased coverage, especially within the potentially sublethal range of exposures to which bees might be exposed in the field, is needed to increase the predictive power of laboratory studies. Attention should be given not only to field-realistic concentrations, but also to feeding regimes, i.e. how the exposure is likely to occur over time in the field. For example, bumble bee colonies that are exposed to a two-week pulse of neonicotinoids simulating oilseed rape flowering, might be able to compensate for this exposure at the colony level through increased brood production if the exposure period is followed by a period of no neonicotinoid exposure [53]. Another way to increase realistic conditions in laboratory studies is to allow bees to forage for their food in the laboratory [76] or to use combined approaches with field realistic exposures of neonicotinoids in the laboratory and free foraging of bees in the field (e.g. [50–51,77–78]).

Field studies were the second most common methodological approach. Several studies measured only pesticide residues in bees or sources in the environment that bees come into contact with and/or ingest. These studies provide important information about field realistic exposure for bees, but the potential to draw further conclusions about the impacts on bees from such data are limited for several reasons. First, concentrations of neonicotinoids that were found are typically well below levels known to cause acute mortality [25], but they might be at levels that cause sublethal effects [27,79]. This complicates the interpretation of residue studies as sublethal effects are not fully characterised, and their consequences more difficult to assess. Second, metabolism of neonicotinoids within hours after ingestion by bees [64,80–82] may lead to an underestimation of exposure based on data from studies that only sample pesticide residues in bees. Third, for studies that measure neonicotinoids in the environment (e.g. in pollen and nectar, and in guttation fluid [41,83]), it often remains uncertain to what degree individual bees and colonies will be exposed to these sources. Finally, residue studies typically provide only a measure of exposure at one point in time (e.g. [84]). The exposure profile over time, i.e. how long bees are affected by varying concentrations of neonicotinoids in the field, typically remains unknown.

In another similar type of field studies, measures of bee exposure to neonicotinoids were related to effects by observational or correlational approaches. For example, measuring residues in bees suspected to be poisoned by pesticides (e.g. [85]) or in weak versus strong honey bee hives (e.g. [86–87]). Twelve field studies used designs in which bee colonies were placed in or near crop fields that were either treated with a neonicotinoid, or not, and then followed different aspects of bee or colony performance [29,48,88–97]. Recently, two such studies have demonstrated negative effects of seed treatments in oilseed rape on *B*. *terrestris* colony performance [29,96]. The study by Rundlöf *et al*. [29] also showed a negative effect on *Osmia bicornis* nesting success next to seed treated oilseed rape. No clear effects of neonicotinoids have been found in any of the studies that measured effects on honey bee colony performance, leading to the conclusion that neonicotinoid seed treatments of these crops are safe for honey bees. However, at least two reservations about this conclusion have been raised. First, given the low number of replicates (fields) and high level of intrinsic variability in performances of honey bee colonies, these field studies have low statistical power to detect any sublethal colony effects [12,29,98]. Second, as Goulson [18] rather skeptically pointed out, it is “essentially impossible to conduct a controlled experiment with free-flying bees.” The bee species studied in these field experiments, *A*. *mellifera* or *B*. *terrestris*, can forage over several kilometres in the landscape [99–100] (for *A*. *mellifera* in extreme cases up to 15 km from the hive [101]). This means that in a typical study landscape, bee colonies in control fields may be exposed to neonicotinoids from other fields that have been treated (see e.g. [102]), while bee colonies in treated fields are expected to also visit non-treated fields. A higher level of replication of fields, larger treated areas of crops per field, efforts to standardize bee colonies, and carefully paired treated and untreated fields in similar landscapes may be able to at least partially overcome these problems for honey bee studies. The current restriction on usage of neonicotinoid seed treatments on crops attractive to bees in the EU provides further opportunities to improve this type of field study, because contamination of control fields is less likely. Such trials should be facilitated by policy and authorities in the EU and member states during the current moratorium period.

A final category of field studies consisted of experiments performed in the field with bees that were artificially fed with neonicotinoids after which bee or colony performances were measured (e.g. [103–105]). This type of field study provides the highest level of control. However, these studies have many similar disadvantages as laboratory studies. Although it may not be straightforward to assess, it is often concluded that exposures are at the higher end, or indeed above, those likely to be experienced in the field [27].

Semi-field studies, with cages in field conditions, were only occasionally used. It is a methodological approach that provides an intermediate level of control between field and laboratory studies. Semi-field studies provide a promising opportunity for further research on the side-effects of pesticides on non-target insects [106]. For example, in a semi-field study entire honey bee colonies were enclosed in 200 m² tunnels on flowering seed treated crops throughout the flowering period [92]. This simulates a realistic worst-case scenario in an intensively managed farmland landscape where an entire colony collects all of its food resources from a seed treated crop during its flowering period. Unfortunately, only residues of neonicotinoids in bees and plants were reported in that study, but it is a promising and evidently tractable approach for future studies if they also include cages on control fields and measure behavioural and life history impacts on the bees and their colonies.

Modelling and risk assessment *in silico* are useful tools for understanding the ecotoxicology of pesticides, especially at higher levels of biological organization [107], but there were few such approaches in the scientific literature on neonicotinoids and bees. Significant regulatory efforts are underway, at least in the EU, to improve risk assessment for neonicotinoid effects on bees [108]. Priorities for the development of risk assessments are to cover the full range of exposure routes and effects that are important for population development and to include bee species other than *A*. *mellifera*. Modelling efforts to predict the effects of neonicotinoids on bees at the colony level are still scarce (but see [109–112]), but represent a promising avenue for future expansion. Bee colony models offer a fast and cost effective alternative to large scale field studies, and they are useful tools to synthesize available knowledge and guide further empirical work [110,113]. Population modelling approaches are informative for evaluating the responses of beneficial insects to pesticide disturbance (e.g. [114]), but we are not aware of any models that have been developed to evaluate the effects of neonicotinoids on bees.

### Biological levels–from sub-individual to population effects

Studies at the sub-individual level can reveal the mode of action of neonicotinoids on bees [115], provide insights on whether bees can detect and regulate their dietary intake of neonicotinoids [116] and give a better mechanistic understanding about how and when neonicotinoids are expected to interact with other pesticides [117] and bee pathogens [118–119]. However, only a minority of studies at this level made direct links to higher levels of organisation by measuring and reporting consequences for individual bees and the colony (S1 Table, but see e.g. [120]). In general, there is a limited understanding of how sub-individual effects of pesticides translate into consequences for the individual, population and community levels [107].

Studies that measure the effects on the individual were most common. They provide insights on the lethal toxicity of neonicotinoids on bees (e.g. [10]), as well as sublethal effects on factors such as longevity, foraging behaviour, feeding, learning and memory (e.g. [62,78,121–123]). Most studies measured the effects on the adult life stage whereas the effects on brood, larval or pupal stages received much less attention. Exposure to neonicotinoids in early life stages of bees can have both direct lethal effects and delayed sublethal effects on the adults [124,125]. Furthermore, chronic toxicity for bees at time points beyond a standard 10 days test time was rarely considered, even though neonicotinoids might cause delayed and time-cumulative toxicity [50,78,126]. A limitation of measuring only the effects at the individual level is that reinforcement or compensatory responses to pesticide exposure at the colony level are not accounted for. In honey bees, bumble bees and other social bees, the colony is the unit of reproduction, and losses of individual bees can be tolerated up to a certain level without consequences for survival or reproduction of the colony [127]. While most bumble bee studies included measurements of colony effects, few of the honey bee studies did (see S1 Table). Nevertheless, the colony level is of primary interest in managed honey bees, because reduced colony performance and higher rates of colony loss directly affect beekeepers. One reason for the relative paucity of colony level studies may be methodological difficulties of performing replicated studies on honey bees, which have large and variable colony sizes and strengths. Future research should therefore try to overcome these difficulties, for example by increasing sample sizes (see e.g. [98]) and employing increased levels of standardization among colonies in order to determine how neonicotinoids affect honey bee colony performance.

We defined population level effects as a change in the population size across at least one completed life cycle of the study organism. According to this definition we found no studies that measured population effects of neonicotinoid exposure on bees, although several studies indicated that such effects might be prevalent. For example, two studies recently showed significantly reduced queen production in bumble bee (*B*. *terrestris*) colonies exposed to neonicotinoids in field settings [29,96]. Further, in one of these studies *O*. *bicornis* did not colonize and reproduce in trap nests placed next to treated fields as compared with control fields [29], again suggesting population level effects. Whether such effects on reproductive success ultimately translate into impacts on the population size of wild bees depend on several factors. These include how reproduction is affected by exposure to neonicotinoids across the bee population on a landscape scale, and not just for bees nesting next to treated fields. It also depends on how negative effects on reproduction, caused by exposure to neonicotinoids, in turn affect density-dependent population regulation, for example through decreased competition for nesting and floral resources or through increased risk for Allee effects [27,128]. Possible ways of overcoming the lack of information on the population effects on wild bees would be to monitor bee populations over several seasons in landscapes with different patterns of neonicotinoid use and/or to use population modelling approaches that are informed by experimental data.

## Conclusions

Based on our systematic literature review we conclude that despite considerable research efforts, there are still significant knowledge gaps concerning the impacts of neonicotinoids on bees. We found that studies were not representative of the diverse and global use of neonicotinoids that are applied in a multitude of insect pollinated crops and non-crop plants that are often visited by multiple bee species. Additionally, we found opportunities for methodological improvements. Laboratory approaches were most common, and we suggest that their capability to infer real-world consequences can be improved by using information from field studies to inform more field realistic exposures to neonicotinoids. Many of the studies with field approaches examined only potential exposure to neonicotinoids, and we suggest that more field studies should measure bee responses to these chemicals. While the majority of studies measured pesticide effects on individual bees, there is a need for more studies that link effects at the individual to mechanisms at the sub-individual level, and also to consequences for colonies and populations. Current research momentum, and recent rapid increases in the number of studies being published on this topic, provide opportunities to provide a more comprehensive understanding of how neonicotinoids are affecting bees. It is important to keep in mind that our review was based only on studies available on Web of Science and PubMed, and that additional information on the effect of neonicotinoids on bees is available in journals not listed in those databases, reports and regulatory pesticide risk assessments. We expect that additional studies from developing countries, individual lethal bee toxicity and risk assessment data might be available from these additional sources. Increased access and use of such data from regulatory pesticide risk assessments by the scientific community has the potential to improve our evidence base concerning the effects of neonicotinoids on bees [27].

Both managed and wild bees are subject to multiple, interacting environmental pressures [3–4,129]. Bees in modern agricultural landscapes are typically exposed to several classes of pesticides [84], creating opportunities for multiple combined effects of pesticide exposure. For example, the effect of neonicotinoids might be additive for bees in combination with other classes of insecticides such as pyrethroids [50,78] or in-hive pesticides [117], or perhaps even interact synergistically with fungicides [10,63], although the field relevance of some of these interactions has been questioned [46,130]. There is also evidence that neonicotinoids can promote additive or synergistic effects when combined with pathogens or parasites of *A*. *mellifera* [118–119,131–134] and *B*. *terrestris* [135]: but the importance of neonicotinoid-pathogen interactions under field-realistic conditions might have been overemphasized [136]. Future research must both disentangle how important neonicotinoid use is relative to other potential drivers of bee declines [3,4], as well as determine the identity and magnitude of interactive effects among these drivers in the field.

## Supporting Information

S1 PRISMA Checklist PRISMA Checklist. (DOC)

S1 Table References and data. (XLS)
